# Transoral Flexible Laser Surgery of the Larynx with Blue Laser

**DOI:** 10.3390/jcm12165250

**Published:** 2023-08-11

**Authors:** Ramón González-Herranz, Mar Martínez-Ruiz-Coello, Estefanía Hernández-García, Estefanía Miranda, Cristina García-García, Oscar Arenas, Guillermo Plaza

**Affiliations:** 1 Department of Otorhinolaryngology, Hospital Universitario de Fuenlabrada, Universidad Rey Juan Carlos, 28042 Madrid, Spain; rgherranz@salud.madrid.org (R.G.-H.); marmruizcoello@hotmail.com (M.M.-R.-C.); fanyhdzgarcia@hotmail.com (E.H.-G.); estefania-miranda@hotmail.com (E.M.); crisgar223@gmail.com (C.G.-G.); osands@hotmail.com (O.A.); 2 Department of Otorhinolaryngology, Hospital Universitario Sanitas La Zarzuela, 28023 Madrid, Spain

**Keywords:** blue laser, laryngeal lesions, photoangiolytic laser, office, operating room

## Abstract

Introduction: Laser surgery of the larynx is currently the standard of clinical practice in a multitude of procedures. Lasers with photoangiolytic properties have a wide application in endolaryngeal lesions. One of their most prominent features is the ability to coagulate blood vessels, reducing unwanted tissue damage. Our objective is to expose the uses of the blue laser (445 nm) in the larynx. Material and methods: A retrospective study was carried out including 47 patients treated with blue photoangiolytic laser from October 2021 to January 2023 at a university hospital. Demographic data, type of lesion presented, date of intervention and scope of the procedure, as well as the parameters of the laser used, were recorded. The number of sessions received per patient, the result and complications were also collected. Results: A total of 47 patients with laryngeal lesions were treated, including vascular angiomas, laryngeal sulcus vocali, vocal cord polyps, Reinke’s edemas, laryngeal papillomatoses, subglottic stenosis, laryngeal synechiae, subglottic granulomas, glottic scars, vocal fold leukoplakias, laryngeal dysplasias and tracheostomal granulomas. The mean age was 52.5 years, and 64.3% of the patients were women. The range of power used in the resective surgeries was 2–10 Watts with a 20-millisecond window. The average number of sessions received was 2.1 (range 1–4). A satisfactory situation was obtained in 45 of the 47 patients treated (95.75%), and an evident decrease in lesions was seen in the remaining two. There was no evidence of any complications directly derived from the use of the blue laser. Twenty-seven cases (54%) were treated exclusively in-office. Conclusions: The blue laser is safe and effective in the treatment of a wide range of laryngeal pathologies. Its advantages include its portability, its photoangiolytic qualities as well as its ability to vaporize tissue in contact mode, which can treat subepithelial vessels or resect lesions.

## 1. Introduction

Laser surgery of the larynx has revolutionized our clinical practice. CO_2_ lasers (wavelength 10,600 nm) used under suspension microlaryngoscopy in general anesthesia are today standard instruments for cutting tissue due to the laser’s property of reducing bleeding during tissue dissection [1,2,3].

**Figure 1:**
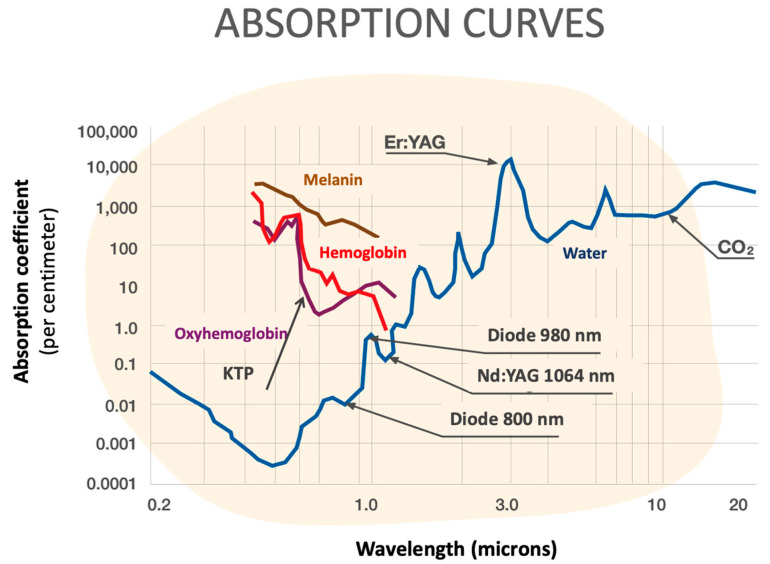
Different wavelengths and absorption coefficients of laryngeal lasers.

Approximately 20 years ago, and as a new parallel field for properties other than laser cutting, photoangiolytic laser surgery was developed. This type of laser included PDL (pulsed dye laser), at a 585 nm wavelength [4,5,6], or KTP (potassium titanyl phosphate), at a 532 nm wavelength [7,8,9] (Figure 1). They routed through flexible glass fibers into flexible channeled endoscopes, expanding the therapeutic armamentarium in a new way by specifically targeting vascularized endolaryngeal lesions.

Among other characteristics, a very advantageous property of photoangiolytic lasers is their ability to coagulate superficial and subepithelial blood vessels with minimal damage to the epithelium between the laser and the lesion, reducing the absorption of energy from the surrounding tissue. Photoangiolytic lasers applied by means of very small caliber fibers (300 or 400 μm) allow for a very selective application of the laser pulses, being very selective on the tissue to be treated. Until the appearance of the blue laser, we were forced to choose between cutting lasers (prototypically a CO_2_ laser) or a photoangiolytic laser (PDL, KTP) [10], but in the case of the blue laser, it combines these two characteristics.

The office laser procedures in laryngology have gained a significant amount of popularity in recent decades using CO_2_, PDL or KTP lasers [11,12], named as Transnasal Flexible Laser Surgery (TNFLS) according to the classification of the European Laryngological Society [13]. This revolutionized practice is attributed to the advent of fiberglass technology and supply of the laser beam. Patients with structural laryngeal disorders who were traditionally treated with direct laryngoscopy and under general anesthesia are now offered laryngeal treatment without sedation in the office.

Recently, the use of a blue photoangiolytic laser (Wolf 445 nm TruBlue A.R.C. Laser, Nürnberg, Germany) has been reported by Hess et al., concluding that the blue laser could operate at least as well as the KTP laser, with the additional possibility of cutting [14,15]. This laser is a class 4 laser [16], which emits energy with a semiconductor within a range of power from 1 miliWatt to 20 Watts and interacts with tissue through 300, 400 and 600 μm fibers. Its absorption rate in the spectral red and black range is approximately 10 times higher than the KTP laser’s. In a single frequency (445 nm), photoangiolytic and cutting properties are combined, characteristics that are unique for a fiber-guided laser (Figure 1).

As Hess et al. [14] concluded, the 445 nm laser features a favorable combination of photoangiolysis and cutting properties with one wavelength at 445 nm. Another major advantage is its property of being transmitted via small glass fibers at 300 and 400 μm, e.g., through a working channel of a transnasally forwarded flexible endoscope. Further advantages are the portability of the shoe box-sized laser and the potential reduction in pulse rates to 1 ms, similar effects on tissue when compared to KTP lasers.

Campos et al. [17] have reported the use of this blue laser on 38 patients under general anesthesia, using direct laryngoscopy techniques (Transoral Flexible Laser Surgery (TOFLS), showing it is a reliable tool to perform minimally invasive surgeries in the operating room using TOFLS techniques. Similarly, Miller et al. [18], Hamdan et al. [19], Ghanem et al. [20], Filauro et al. [21] and Hamdan et al. [22,23] presented several case series of patients treated with in-office blue laser (TNFLS), concluding that this tool is effective for many laryngeal pathologies.

The advantages of this new technology comprise machine transportability and a notable combination of photoangiolytic and cutting properties that can be adjusted by modulating the distance-to-target of the fiber’s tip. Moreover, this tool will increase the possibilities to treat laryngeal pathology in the office, reducing delays and allowing for complex cases to be addressed in a straightforward manner.

The objective of this study is to report our results with a similar new blue photoangiolytic laser (MEDICAL INN SPAIN, Barcelona, Spain) on laryngeal pathology discussing its potential uses, safety issues, limitations, risks and possible complications.

## 2. Materials and Methods

This paper presents a case series of patients who were treated with a fiber-guided photoangiolitic blue laser (445 nm) (Intermedic, Barcelona, Spain) including 47 patients whose reports were collected from October 2021 to January 2023 in a university hospital, having performed 99 procedures in the consultation or operating room.

Demographic data, type of lesion presented, date of intervention and setting of the procedure (office or operating room) were recorded, as well as the parameters of the laser used to treat the lesions. The number of sessions received by each patient, the result obtained and the complications observed during treatment were also collected.

The inclusion criteria were patients older than 18 years with laryngeal lesions (supraglottic, glottic and subglottic) that required resection, including in this group exudative lesions from Reinke’s space or subglottic lesions that implied respiratory compromise, as well as structural lesions such as scars or sulcus vocalis that limit the mucosal wave by fixing the epithelium to deep layers of the vocal cord and that are susceptible to endoscopic treatment in the office or operating room. Patients with hematological diseases or coagulation disorders were excluded.

When proposing this therapeutic option compared to other commonly used options, the possible risks and advantages offered using the blue laser were explained to the patient, expressly including it as an addition to the informed consent corresponding to each pathology.

The minimum follow-up of the patient was 4 months, presenting a range from 4 to 24 months, adapting to each pathology treated, with resolved pathologies being discharged without probable recurrence, and long-term follow-up of lesions susceptible to having a worsening or reappearance as in the case of subglottic stenosis or papillomatosis.

As this is a descriptive study of the lesions treated by blue laser, we have not carried out statistical analysis as it is not the objective of this study, and said lesions are very diverse pathological entities.

In procedures performed under general anesthesia, the fiber was introduced through a laryngoscope attached to a microclamp commonly used for laryngeal microsurgery, through a direct laryngoscopy aspirator, or attached to a 0 or 30 degree endoscope (see Supplementary Videos S1–S3).

**Figure 2:**
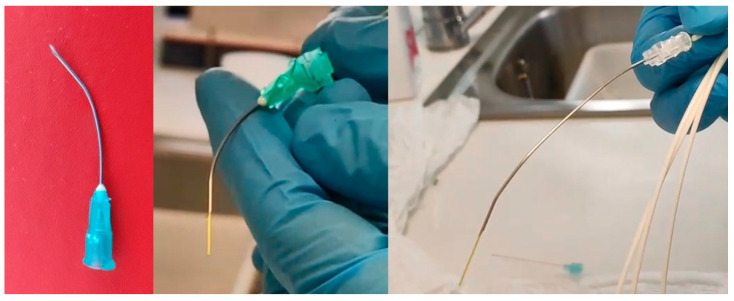
Needle to perform percutaneous blue laser procedures through the cricothyroid or thyrohyoid route.

**Figure 3:**
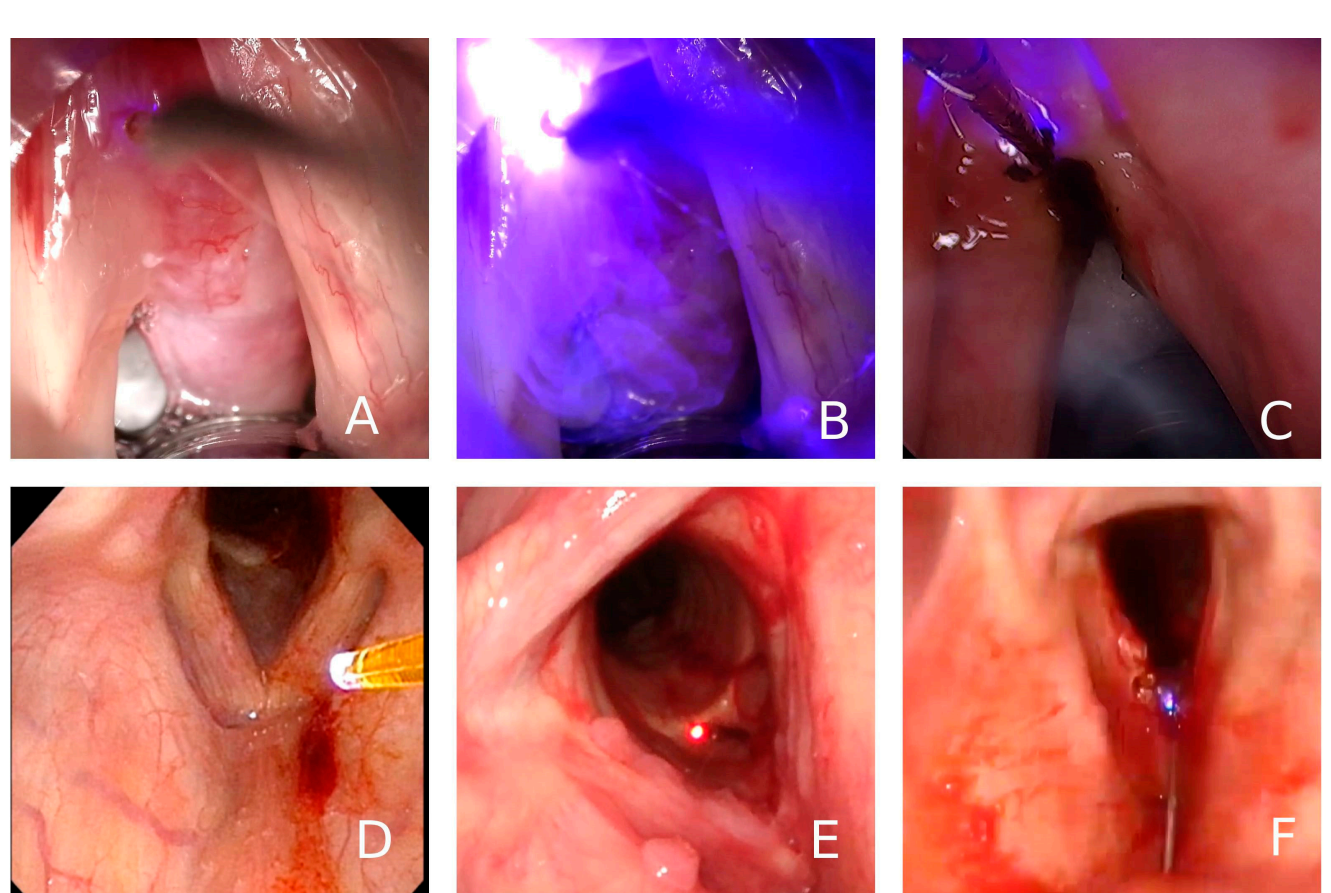
Modes of use of the blue laser under general and local anesthesia as treatment of laryngeal lesions. (**A**) Large laryngeal polyp approached by direct laryngoscopy under general anesthesia, (**B**) Action of the laser in contact mode in the same patient, (**C**) image of the lesion already resected, (**D**) use under local anesthesia by channel videofiberscope, (**E**) passage of the fiber through a needle by cricothyroid approach (**F**) and by thyrohyoid approach.

In procedures performed under local anesthesia, the fiber was introduced through a canal videofibroscope, or through a needle to perform percutaneous procedures through the cricothyroid or thyrohyoid route, while controlled with a transnasal videofiberscope (Figure 2). In Figure 3, we can see different ways of drive the fiber though a direct laryngoscopy or under local anesthesia with a videofibroscope or though transcervical puncture.

The approval of the hospital’s ethics committee was obtained to perform this retrospective review (IRB: APR 23/12).

## 3. Results

Forty-seven patients with laryngeal lesions were treated with blue photoangiolytic laser (Table 1). The laryngeal pathologies treated were papillomatosis, dysplasia/leukoplakia, vascular angioma, subglottic granuloma, subglottic stenosis, tracheostoma granuloma, laryngeal synechia, scar/sulcus/vergeture, vallecular cyst, vocal cord polyp and Reinke’s edema. The mean age was 51.2 years, and 64.3% of the patients were women.

The most used laser fiber size was 300 nm (range 300–600). The power used in resective surgeries (granulomas, synechiae or polyp) was 5–10 W with a 20–50 millisecond (ms) window and a 100 ms rest. It should be noted that, for vascular lesions and scars, we used the indirect way (3–5 mm distance) to eliminate them. In addition, in these lesions the power required was lower (2–3 W), but the number of sessions received was three sessions on average. The work intervals when trying to eliminate scars or vascular lesions were 10 ms and the rest were 50–200 ms. The sessions were carried out with a week’s interval until a sufficient improvement of the lesion was achieved.

The average number of sessions received in general was 2.1 (range 1–4). The lesions that required the higher number of sessions were scars, laryngeal papillomatosis and granulomas (average of three sessions received). Laryngeal lesions that were treated in a single session were laryngeal synechiae, vocal cord polyps, Reinke’s edema and leukoplakias, as these were resection procedures.

Of the 47 cases, 54% (27/47) were treated exclusively in consultation, 20% were treated in the operating room and 26% required a combination of consultation and operating room.

A satisfactory situation with a resolution of the laryngeal lesion was obtained in 45 of the 47 patients treated (95.75%), and an evident decrease in lesions in the remaining two. In one patient with laryngeal papillomatosis, bevalizumab was administered simultaneously in the bed of the lesions, to improve the control. A patient with a laryngeal scar underwent a first session in the operating room and a second session in the office, which was poorly tolerated by the patient, and subsequent sessions were not scheduled.

Regarding complications, there was no evidence of any complication directly derived from the use of the blue laser. One of the patients with laryngeal granuloma presented an increase in the size of the granuloma after the first procedure, attributing it to a partial treatment of the lesion due to its volume. The patient had to go to the emergency room due to dyspnea and she was treated with corticosteroid to improve. The patients with laryngeal synechia and subglottic stenosis were diagnosed with COVID-19 the week after the procedure in the emergency room of our hospital before receiving the second laser session in the office, presenting a worsening of the clinical situation due to this disease. These clinical worsening of the patients is not attributable to the procedure used for their treatment, but to the clinical situation of the pandemic and the natural evolution of the treated lesions themselves.

**Figure 4:**
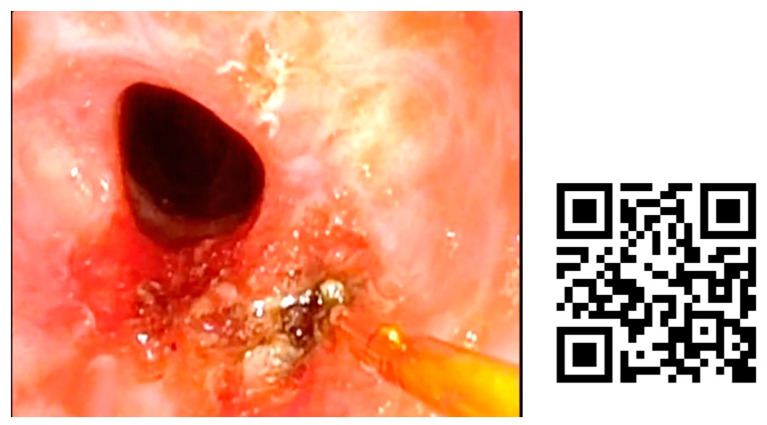
Subglottic stenosis treated by blue laser in ENT office. She was diagnosed after presenting to the emergency department for progressive dyspnea of 10 days’ evolution. As a relevant history, the patient had been admitted to the ICU for 30 days, 15 days with orotracheal intubation. Fibroscopy examination was performed to diagnose the stenosis. After anesthetizing the larynx by subglottic infiltration of local anesthetic, a channel videofibroscope was used and a 400 nm fiber was employed to perform radial resection of the subglottic stenosis, without further topical treatment.

In Figure 4, we present an example of subglottic stenosis after a long intubation in the ICU. The patient always remained stable and blue laser treatment was performed in the office.

**Figure 5:**
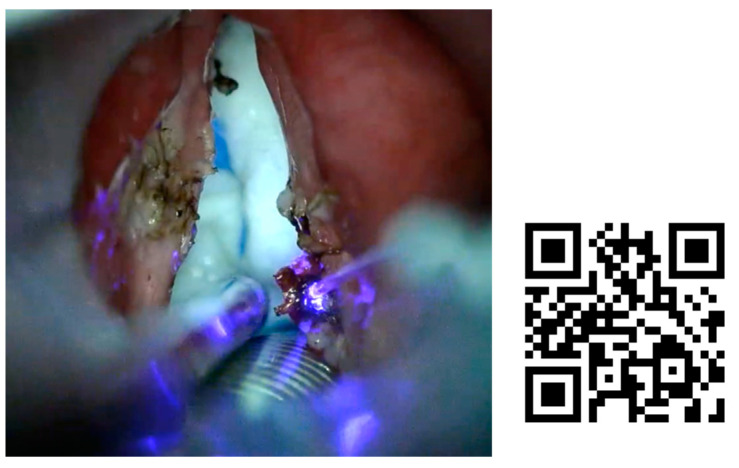
Laryngeal papillomatosis treated in the operating room with blue laser. A 34-year-old man with laryngeal papillomatosis in ENT follow-up for two years, presenting papillomatous lesions in the glottis and supraglottis. It was decided to treat these lesions in the operating room, to take a biopsy of the lesions and treat all visible lesions. Direct laryngoscopy and excision of the lesions was performed, using blue laser. After the intervention, bevalizumab was applied to the bed of the lesions to improve their control.

In Figure 5 can be seen a patient who came back after two years without lesions of papilloma, presenting new lesions in the glottis and supraglottis. We decided to treat under general anesthesia, to confirm diagnosis by a biopsy.

**Figure 6:**
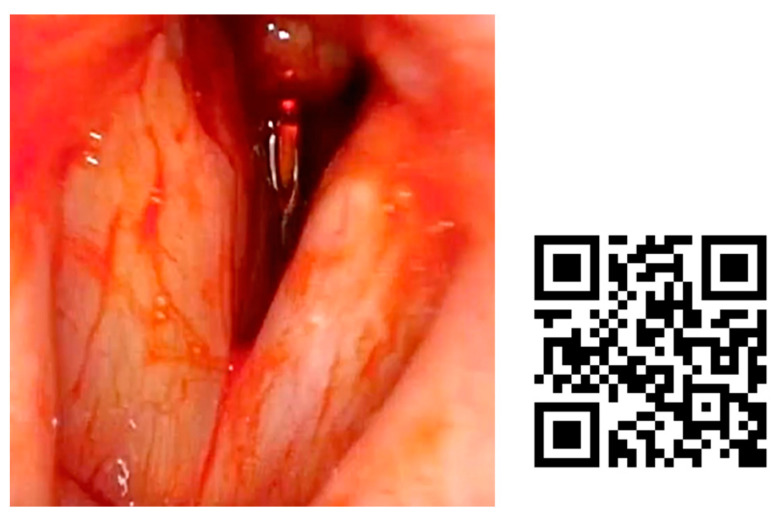
Treatment of laryngeal granuloma with blue laser in consultation room. A 68-year-old woman with dyspnea that had lasted for days and a sensation of a foreign body in the larynx. She was admitted to the ICU for two months, with orotracheal intubation for 1 month. The high dyspnea had progressed until it became resting. The patient presented with stridor and, by fibroscopic examination, two subglottic granulomas and a laryngeal synechia were found that obstructed the lumen by more than 50%. The lesions were treated in the consultation room with blue laser.

**Figure 7:**
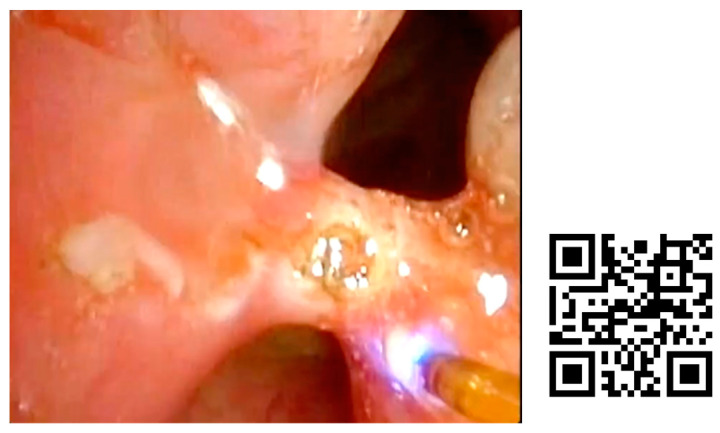
Posterior glottic mucosal bridge treatment with blue laser in consultation room.

A case of granuloma and posterior commissure mucosal bridge is shown in Figure 6 and Figure 7. After orotracheal intubation for 1 month, a high dyspnea had progressed. The patient presented with stridor and, by fibroscopic examination, two subglottic granulomas and a laryngeal synechia were found.

## 4. Discussion

This study shows that the blue laser is a very useful tool in laryngology, allowing for treatment from subglottic stenosis to less compromised lesions such as laryngeal polyps or Reinke’s edema. It has allowed us to move to the office patients requiring rapid treatment in situations of airway compromise during the COVID-19 pandemic. It has streamlined the management of these patients, while we have given an alternative option to the more usual treatment under general anesthesia.

The fact that this is a new alternative tool in our hands has not compromised the safety of patients or the results obtained, having facilitated on many occasions the agility and shortening of action times at a time when accessibility to procedures under general anesthesia in an operating room was more limited than usual. The learning curve carried out during this period has allowed us to define the power protocols and times of action and rest of the laser in each procedure, as well as the effects of the laser on the different tissues.

The irruption of photoangiolytic lasers in otorhinolaryngological practice has mainly improved the possibility of using the laser to treat laryngeal lesions under local anesthesia, moving these procedures from the operating room to the office [12,13]. On the other hand, the angiolytic characteristics of the blue laser and its characteristic absorption by the tissues result in a reduction in mucosal lesions [14,15,16,17]. Thanks to this, it can be used to perform tissue resections, greatly limiting the affected neighboring tissue, even when performed with the patient awake under local anesthesia [3].

The results of this investigation agree with published reports on blue laser therapy for vocal fold lesions. Several papers have presented case series of patients treated with blue laser in-office such as Miller et al.’s [18], Hamdan et al.’s [19], Ghanem et al.’s [20], Filauro et al.’s [21] and Hamdan et al.’s [22,23]. They have demonstrated the usefulness of this laser resecting laryngeal lesions in consultation in awake patients. They have shown good results in lesions of the free edge of the vocal cord, as well as in papillomatosis, having been able to transfer these procedures traditionally performed in the operating room to the office. In our practice, this has meant a great change, shortening times and simplifying the patient’s stay in the hospital, especially in recurrent procedures such as laryngeal papillomatosis. In our series, we have expanded the indications, not limiting ourselves to lesions of the free edge of the vocal folds, having treated, in consultation, patients with more complex pathologies such as angiomatous lesions, scars or even subglottic stenosis.

Campos et al. [17] used the blue laser as a new tool to treat patients with pathologies distal to the glottic plane, such as subglottic stenosis. This group has demonstrated its efficacy under general anesthesia and direct laryngoscopy, associating it with other treatment modalities such as balloon dilatation or CO_2_ laser resection. In our series, we have managed five patients with subglottic stenosis with blue laser, and most of them in consultation, all of them requiring several treatment sessions, but with a satisfactory and lasting result. As in the series of Campos, we have been helped in this type of lesions by associated treatments such as mitomycin.

Balouch et al. [24] compared the 532 nm potassium–titanyl–phosphate (KTP) laser and the 445 nm blue laser for treating vascular malformations of the vocal cords. The blue laser was shown to have the highest absorption rate by hemoglobin, so it can be used at lower powers to minimize thermal injury to adjacent tissue. Edema and hemorrhage of the vocal cords were shown to significantly decrease post-operatively when treated with a blue laser rather than with a KTP laser. Lin et al. [25] compared the blue laser and the KTP laser regarding the degree of scarring presented after treatment of the vocal folds of a normal rat. Their results showed a significant increase in fibrosis in the vocal folds, as well as an earlier presentation of this phenomenon after treatment with a KTP laser, and no evidence of this in the blue laser cases.

The variety of lesions treated in our study, as well as the combination of both in-office and in the operating room conditions, have allowed us to gain valuable experience regarding managing the necessary powers for each type of lesion to be treated, as well as the different combinations of work and rest windows. This has also been extensively shown in a recent book on blue laser in laryngology, published by Hamdan et al. [26]

Another important factor when using this laser is the effect on the tissue depending on the distance from the fiber tip to it and the ease of use and manipulation of the different fibers. In this sense, the fact of increasing the caliber of the fiber used allows for a greater rigidity of it, which makes it ideal in procedures by direct laryngoscopy, joining it to a forceps or laryngeal microsurgery aspirator to facilitate the handling of the laryngeal microsurgery. However, the finest fibers are ideal when seeking precision performance or when it is required to pass the fiber through a working channel or a percutaneous needle.

At the treatment stage of the transcutaneous approach to reach the larynx in the office, the passage of the fiber needs to be performed through a needle that must be bent to facilitate better access to the lesions. The use of larger gauge fibers forces us to use 16 G gauge needles, worsening handling and making it difficult to bend without breaking the fiber that we have put inside. This is the reason why the most used fibers in office procedures are 300 and 400 nm.

Another advantage seen with the blue laser has been the optimal management of bleeding during procedures. This is especially observed in procedures performed under general anesthesia in which extensive tissue resections are performed (see Figure 2), and bleeding is very limited in those performed under local anesthesia.

A possible source of bleeding are the transcutaneous punctures that we use via the access route to reach the lesion to be treated, or the fact that, in patients in whom a channel fiberscope is used, may present erosions in the mucosa when the tip of the fiber injures the patient’s mucosa when swallowing involuntarily.

In our experience, we avoid the use of the laser in continuous mode due to the difficulty in avoiding unwanted tissue damage. For this reason, we use always pulsed mode. The rest windows are, generally, at least two times the activation time of the laser, to avoid overheating of the tissue.

Regarding the complications of these procedures, they have not been notable, being attributable to the methods of anesthesia on the larynx in procedures under local anesthesia, rather than the use of the blue laser itself.

This study has its limitations. One of them is its retrospective nature, and the second is the limited number of subjects. A larger cohort is needed to evaluate the impact of patients’ demographic characteristics and disease specificity. Another limitation is the relatively short follow-up, which does not allow us to draw any conclusion on the disease-free interval following surgery and the long-term recurrence rate.

## 5. Conclusions

The blue laser is safe and effective in the treatment of a wide range of laryngeal pathologies. The key advantages of the blue laser include its portability and its unique combination of photoangiolytic and ablation qualities that make it unique compared to the traditionally used CO_2_ laser and KTP laser. The blue laser is a safe and effective alternative treatment modality to treat laryngeal lesions. Larger numbers of patients and long-term follow-up are needed to better assess the efficacy of this treatment, which has already shown great promise.

## Figures and Tables

**Table 1:** Laryngeal lesions treated with blue photoangiolytic laser. ms: miliseconds; W: watt; GA: general anesthesia; LA: local anesthesia.

Pathology	Cases (*n* = 47)	Age (Mean)	Work Window	Rest Window	Power	Fiber Used	Local vs. General Anesthesia
Papillomatosis	6	46.6	50 ms	150 ms	8 W	400	First GA Follow LA
Dysplasia/Leukoplakia	4	62.7	50 ms	150 ms	4 W	300	GA
Vascular angioma	5	45.5	20 ms	200 ms	3 W	300	GA/LA
Subglottic granuloma	3	58.3	20 ms	100 ms	6 W	400	LA
Subglottic stenosis	5	69.4	30 ms	100 ms	8 W	400/600	GA/LA
Tracheostoma granuloma	3	66	50 ms	100 ms	8 W	400	LA
Laryngeal synechia	4	68	20 ms	100 ms	10 W	400/600	GA
Scar/Sulcus/Vergeture	6	58.5	10 ms	50 ms	2 W	400	LA
Vallecular cyst	1	57	20 ms	50 ms	10 W	600	GA
Vocal cord polyp	7	40.6	50 ms	100 ms	5 W	400	LA
Reinke’s edema	3	52	20 ms	50 ms	10 W	600	GA

## Data Availability

Not applicable.

## Supplementary Materials

The following supporting information can be downloaded at: https://www.mdpi.com/article/10.3390/jcm12165250/s1, Video S1: Reinke’s edema; Video S2: Vocal fold polyp; Video S3: Bilateral vocal fold polyps.
